# Atractylenolide I alleviates the experimental allergic response in mice by suppressing TLR4/NF-kB/NLRP3 signalling

**DOI:** 10.1515/biol-2025-1143

**Published:** 2025-08-08

**Authors:** Weiran Cai, Zhijun Zhang, Chenyan Shi, Ru Sun, Han Ju, Xuelin Dong, Lei Teng

**Affiliations:** Department of Otolaryngology, Shuguang Hospital Affiliated with Shanghai University of Traditional Chinese Medicine, 528 Zhang Heng Road, Shanghai, 201203, China

**Keywords:** allergic rhinitis, atractylenolide I, TLR4, NF-κB, NLRP3, Th1/Th2 imbalance

## Abstract

Allergic rhinitis (AR) is a frequent respiratory condition characterized by elevated immunoglobulin E (IgE) levels and nasal mucosal inflammation. Atractylenolide I (ATL-I), a bioactive ingredient in medicinal plants, is known for its ability to alleviate tissue damage by inhibiting inflammatory and oxidative stress responses. In this study, we aimed to investigate the protective roles of ATL-I in AR and reveal the potential mechanism involved. The AR model was developed in mice by intraperitoneal sensitization followed by intranasal exposure to ovalbumin. The effects of ATL-I on allergic responses were evaluated by recording sneezing and rubbing frequencies and measuring the serum concentrations of Th1 and Th2 cytokines following the intragastric administration of ATL-I. The activation of the Toll-like receptor 4/nuclear factor κB (TLR4/NF-κB) pathway and the NOD-like receptor 3 (NLRP3) inflammasome was assessed via Western blotting and immunohistochemistry. The results showed that ATL-I administration alleviated allergic responses in AR mice, as evidenced by significant decreases in the frequencies of sneezing and rubbing and in the serum concentrations of histamine and IgE. Compared with control mice, AR mice presented downregulated Th1 cytokines and upregulated Th2 cytokines, whereas the Th1/Th2 imbalance was improved by ATL-I. ATL-I reduced the mucosal layer thickness and alleviated goblet cell hyperplasia in AR mice. Furthermore, ATL-I inhibited TLR4/NF-κB pathway activation in mucosal tissues, which resulted in the inactivation of the downstream NLRP3 inflammasome. In summary, our results indicate that ATL-I alleviates allergic responses by inhibiting the TLR4/NF-κB/NLRP3 pathway, providing a promising therapeutic strategy for AR.

## Graphical abstract

**Figure:**
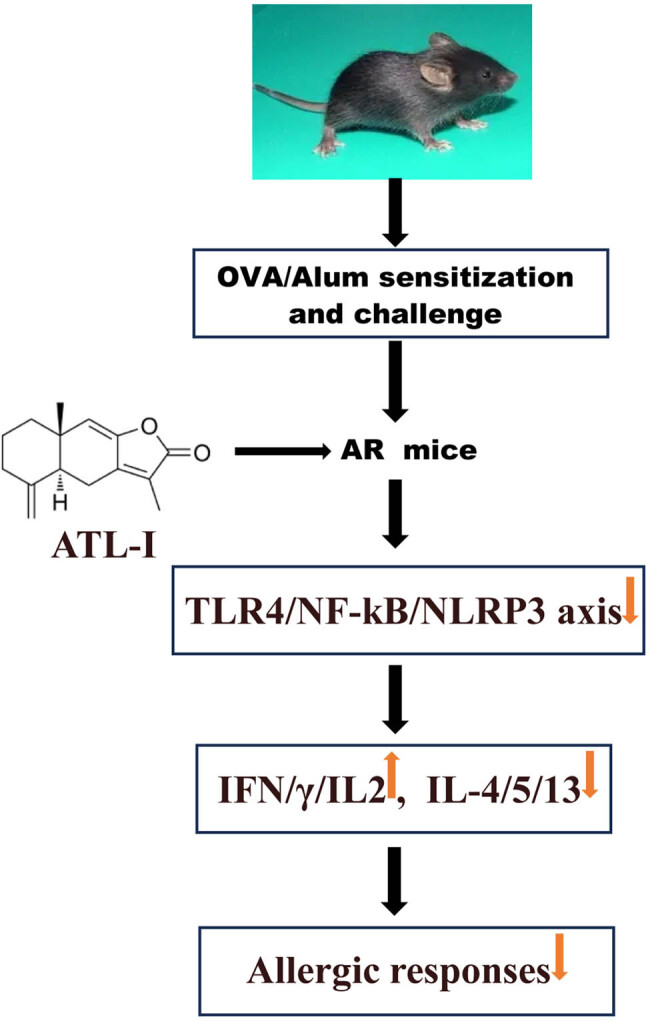

## 1 Introduction

Allergic rhinitis (AR) is a long-term allergic respiratory condition caused by exposure to specific allergens and is regulated by specific immunoglobulin E (IgE) [1]. The condition is frequently diagnosed in rhinology, affecting nearly 20–30% of the population worldwide [2]. AR causes no threat to life but results in nasal itching, sneezing, and rhinophobia, resulting in a reduction in individuals’ quality of life [3]. Currently, the primary medications for AR therapy include H1-antihistamines, corticosteroids, and allergen immunotherapy [4]. Although current treatment methods have made significant progress, no method that can fundamentally cure patients with AR exists. The identification of effective anti-AR drugs and the development of corresponding medical devices are indispensable.

Emerging evidence has shown that plant-derived compounds such as curcumin [5], shenqi [6], and luteolin [7] exert potent protective effects against AR, suggesting that these natural active ingredients might be potential therapeutic strategies for AR. Atractylenolide (ATL) is a member of a class of sesquiterpene lactones that is primarily derived primarily from *Atractylodes macrocephala* [8]. This class encompasses ATL-I, ATL-II, and ATL-III, all of which have demonstrated therapeutic potential. For example, ATL-III has been utilized to treat nonalcoholic fatty liver disorders, spinal cord injuries, and osteoarthritis [9,10,11]. ATL-II has shown efficacy in inhibiting lung cancer cell metastasis and in mitigating radiation damage [12,13]. ATL-I, the principal biologically active component of ATL, is associated with a variety of pharmacological activities. It has been found to protect against breast cancer tumorigenesis via the Toll-like receptor 4 (TLR4)/nuclear factor kappa B (NF-κB) pathways [14] and to suppress colorectal cancer by inducing apoptosis and inhibiting glycolysis [15]. Furthermore, ATL-I has shown promise as a therapeutic agent for managing Parkinson’s disease [16]. However, the role of ATL-I in AR remains to be elucidated.

A T helper 1 (Th1)/Th2 cell imbalance is closely correlated with the onset of allergic disorders, particularly AR [17]. Exposure to an allergen triggers an immune response, leading to Th2 cell proliferation. Activated Th2 cells release cytokines such as IL-4, IL-5, and IL-13, which prompt IgE antibody generation. When re-exposed to the allergen, mast cells and basophils that bind IgE produce inflammatory substances such as histamine, culminating in AR [18]. Conversely, Th1 cells produce interferon gamma (IFN-γ), which inhibits the production of Th2 cytokines [19]. Thus, addressing the Th1/Th2 imbalance may represent a promising approach for the treatment of AR. Excessive activation of the TLR4/NF-κB pathway has a pivotal effect on the Th1/Th2 imbalance. TLR4 activates NF-κB signalling, which subsequently accelerates the expression of proinflammatory cytokines and chemokines [20]. Notably, a recent study revealed that luteolin ameliorates the Th1/Th2 imbalance by inhibiting the activation of the TLR4/NF-κB pathway in AR rats [7], suggesting that TLR4/NF-κB signalling is a potential target for reversing the Th1/Th2 imbalance. Furthermore, NF-κB is a key initiator for activating the NOD-like receptor 3 (NLRP3) inflammasome by increasing NLRP3 expression and triggering caspase 1 activation [20,21]. Activated caspase-1 processes pro-IL-1β and pro-IL-18 into their biologically active forms (IL-1β and IL-18), driving inflammation. It also cleaves gasdermin D (GSDMD), initiating pyroptosis, a distinct form of programmed cell death [22]. The NLRP3 inflammasome plays a vital role in mediating allergic responses [23]. Given the effects of ATL-I on alleviating NLRP3 inflammasome activation in many types of diseases [24,25,26], we aimed to investigate whether ATL-I alleviates allergic responses by inhibiting TLR4/NF-kB/NLRP3 activation in an experimental AR model.

## 2 Materials and methods

### 2.1 Reagents

Ovalbumin (OVA, Cat# HY-W250978), aluminium hydroxide (Cat# HY-B1521), ATL-I (Cat# HY-N0201), and dexamethasone (Dex, Cat# HY-14648) were obtained from MCE (NJ, USA). Enzyme-linked immunosorbent assay (ELISA) kits for mouse histamine (ab213975), IL-2 (ab100706), IL-5 (ab100711), and IL-13 (ab100700), anti-rabbit secondary antibodies (HRP, ab6721), and DAB reagents (ab64238) were obtained from Abcam (MA, USA). ELISA kits for mouse IFN-γ (P1508) and IL-4 (PI612), TRIzol reagent (R0016), RIPA lysis buffer (Cat# P0013B), the BCA kit (Cat# P0012), BeyoECL Plus (Cat# P0018S), the haematoxylin and eosin (H&E) kit (C0105S), and the periodic acid–Schiff (PAS) kit (C0142S) were obtained from Beyotime (Shanghai, China). An IgE ELISA kit (Cat# CSB-E08914) was obtained from Cusabio (Wuhan, China). The cDNA reverse transcription reagent (Cat# 4368814), TLR4 polyclonal antibody (Cat# 48-2300), NLRP3 antibody (Cat# MA5-23919), cleaved caspase-1 antibody (Cat# PA5-99390), GSDMD antibody (Cat# PA5-116815), and phosphorylated p65 (p-p65) polyclonal antibody (Cat# 44-711G) were obtained from Thermo Fisher (MA, USA). The SYBR green mixture was obtained from TaKaRa (Tokyo, Japan).

### 2.2 Animal experiments

The work was conducted with approval from the Animal Ethics Committee of Shanghai University of Traditional Chinese Medicine (approval number: PZSHUTCM211227022) in accordance with the ARRIVE guidelines to minimize animal suffering and numbers. Female C57BL/6 mice (weighing 23–28 g; aged over 8 weeks) were obtained from Shanghai Vital River (Shanghai, China) and maintained in a temperature- and humidity-controlled environment. The AR model was developed by intraperitoneal sensitization with 50 μg of OVA and 5 mg of aluminium hydroxide on Days 1, 7, and 14, followed by intranasal exposure to OVA (20 μL, 40 mg/mL) from the 22nd to 29th days, as previously described [23,27,28]. In accordance with a previous study [24] and our preliminary experiment, AR mice were treated by intragastric delivery of ATL-I (0, 25, or 50 mg/kg) from the 22nd to 31st days. A positive control, Dex (3 mg/kg), was administered via intraperitoneal injection according to the manufacturer’s instructions. Similarly, TLR4 antagonist TAK-242 (MCE, 3 mg/kg) was also administered via intraperitoneal injection. All the animals were categorized into five separate groups (*n* = 6): (i) the Ctrl group, (ii) the AR group (AR mice treated with 0 mg/kg ATL-I), (iii) the ATL-25 group (AR mice treated with 25 mg/kg ATL-I), (iv) the ATL-50 group (AR mice treated with 50 mg/kg ATL-I), and (v) the Dex group. The frequencies of sneezing and rubbing were recorded over a period of 20 min on the 33rd day by two blinded observers to evaluate the severity of nasal symptoms. Nasal mucosal tissues were subsequently collected from all the mice after euthanasia by pentobarbital overdose and cervical dislocation.


**Ethical approval:** The research related to animal use has been complied with all the relevant national regulations and institutional policies for the care and use of animals and has been approved by the Animal Ethics Committee of Shanghai University of Traditional Chinese Medicine (No. PZSHUTCM211227022).

### 2.3 ELISA

The concentrations of histamine, IFN-γ, IL-2, IL-4, IL-5, IL-13, and OVE-specific IgE were measured with commercial kits according to the manufacturers’ instructions.

### 2.4 Quantitative real-time PCR (qRT-PCR)

RNA was extracted from nasal mucosal tissues using TRIzol reagent and reverse transcribed into cDNA with a reverse transcription. qRT-PCR was conducted on a LightCycler 480^®^ PCR system (Roche, Switzerland) in a 10 µL reaction mixture including cDNA, SYBR Green mix, and primers (Table S1). The mRNA level was determined with the 2^−ΔΔCT^ formula after normalization to β-actin [29].

### 2.5 Western blot

Protein extraction was performed using RIPA buffer, the protein concentrations were quantified by the BCA method, and proteins were separated on 12% sodium dodecyl sulfate-polyacrylamide gel electrophoresis gels and subsequently transferred to polyvinylidene fluoride membranes. Afterwards, the membranes were incubated with primary antibodies for 1 h at room temperature, followed by a 60 min incubation with the secondary antibodies. The immunoblots were visualized using ECL reagents and quantified with ImageJ (NIH, MA, USA).

### 2.6 Immunohistochemistry (IHC)

Nasal tissues fixed with 4% paraformaldehyde were embedded in paraffin and sectioned into 4 μm thick slices. After deparaffinization, rehydration, antigen retrieval, and treatment with hydrogen peroxide, the slices were subjected to incubation with primary antibodies for 60 min, followed by a 60 min incubation with the secondary antibodies. After being visualized with DAB reagent and restained with haematoxylin, the slices were imaged with a fluorescence microscope (Olympus, Tokyo, Japan).

### 2.7 H&E and PAS staining

Nasal tissues embedded in paraffin were cut into 4 μm thick slices. Following deparaffinization and rehydration, the slices were stained via H&E and PAS kits according to the manufacturers’ instructions. The slices were photographed using a DM500 optical microscope (Leica, Wetzlar, Germany).

### 2.8 Statistics

The data, derived from three separate trials, are presented as the means ± standard deviation. One‐way analysis of variance (Dunnett’s post hoc test) was applied for multigroup comparisons with the aid of SPSS 20.0 (IBM, NY, USA). A *p*-value of less than 0.05 was considered statistically significant.

## 3 Results

### 3.1 ATL-I alleviated the clinical symptoms of AR mice

**Figure 1:**
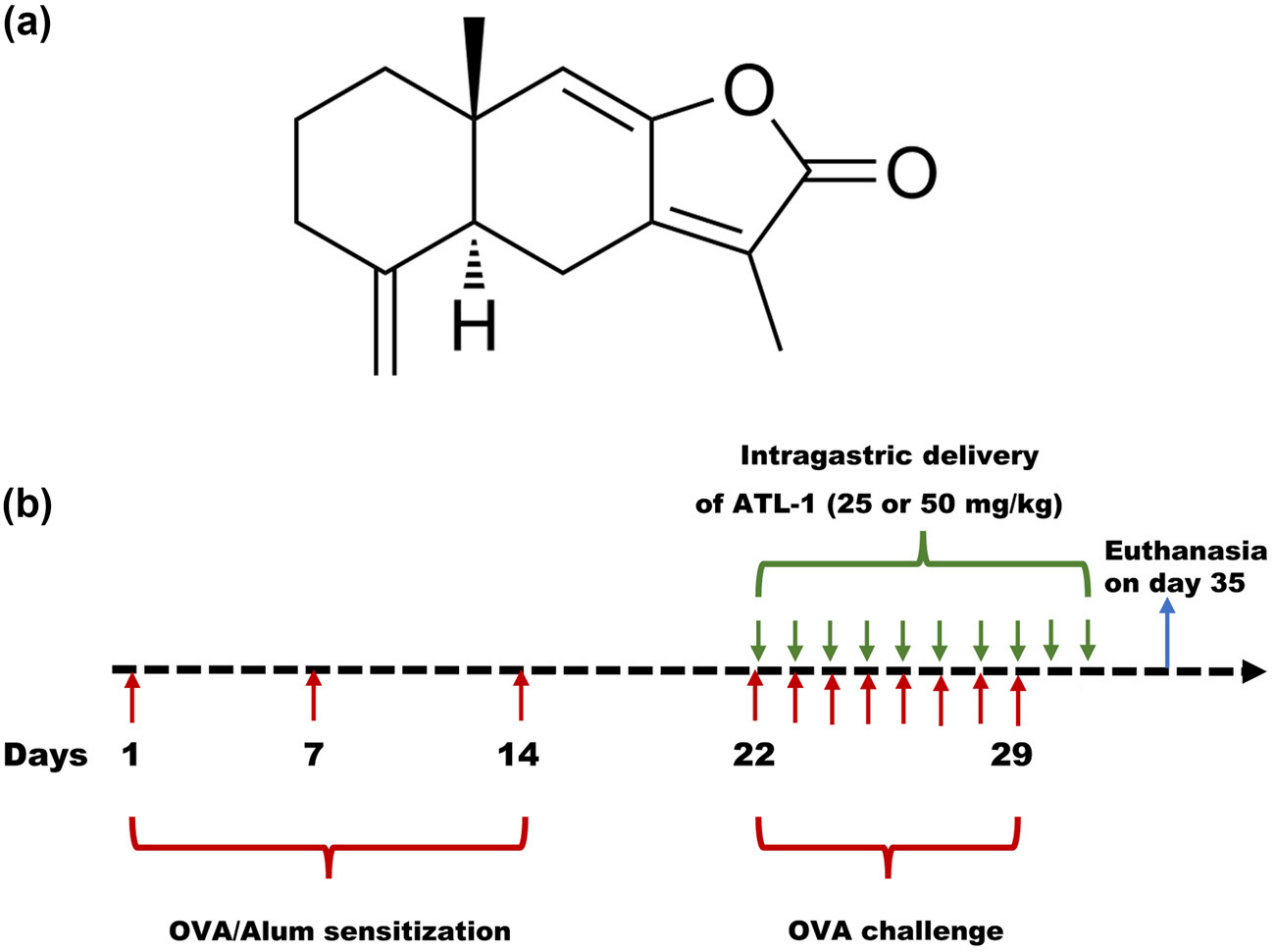
Schematic diagram of the experimental protocol. (a) Chemical structure of ATL-I. (b) Schematic diagram of AR mouse model construction and ATL-I treatment (*n* = 6 mice per group). The AR model was developed in mice by intraperitoneal sensitization with OVA (50 μg) and aluminium hydroxide (1 mg), followed by intranasal exposure to OVA (40 mg/mL) at the time points indicated by the red arrows. The control animals were administered saline. AR mice were treated by the intragastric delivery of ATL-I (0, 25, or 50 mg/kg) or a positive control, Dex (3 mg/kg), at the time points indicated by the green arrows.

**Figure 2:**
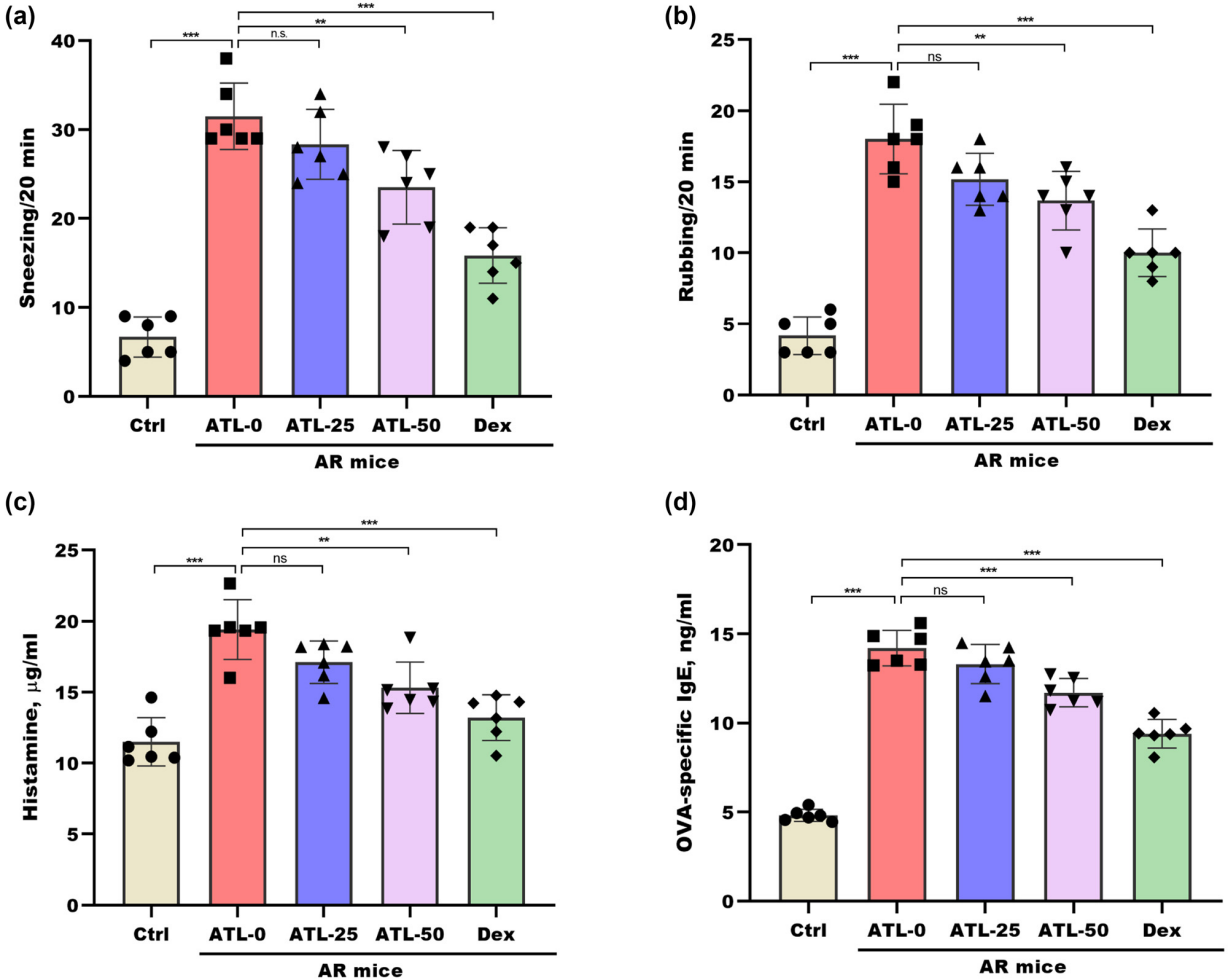
ATL-I alleviated clinical symptoms in AR mice. Sneezing (a) and rubbing (b) frequencies were counted for 20 min in control mice (Ctrl group) and AR mice treated with 0 mg/kg ATL-I (ATL-0 group), 25 mg/kg ATL-I (ATL-25 group), 50 mg/kg ATL-I (ATL-50 group), or 3 mg/kg Dex (Dex group). The serum concentrations of histamine (c) and OVA-specific IgE (d) were measured by ELISAs in the Ctrl group, the ATL-0 group, the ATL-25 group, the ATL-50 group, and the Dex group. Control mice and AR model mice were treated with 0 mg/kg ATL-I (ATL-0), 25 mg/kg ATL-I (ATL-25), 50 mg/kg ATL-I (ATL-50), or Dex (3 mg/kg). *p < 0.05, **p < 0.01, ***p < 0.001, ns, not significant.

An AR model was developed in mice by intraperitoneal sensitization followed by intranasal exposure to OVA to investigate the protective effect of ATL-I (Figure 1a) on AR. Nasal symptoms were measured after the intragastric administration of ATL-I (0, 25, or 50 mg/kg) or a positive control, Dex (3 mg/kg) (Figure 1b). Compared with those of control mice, the frequencies of sneezing and rubbing were significantly increased in AR mice, whereas these increases were significantly reversed by 50 mg/kg ATL-I (Figure 2a and b). In addition, the serum histamine concentration was increased in AR mice compared to control mice, whereas this change was significantly blocked by 50 mg/kg ATL-I (Figure 2c). ATL-I also decreased the serum concentrations of IgE in AR mice (Figure 2d).

### 3.2 ATL-I improved the Th1/Th2 imbalance in AR mice

**Figure 3:**
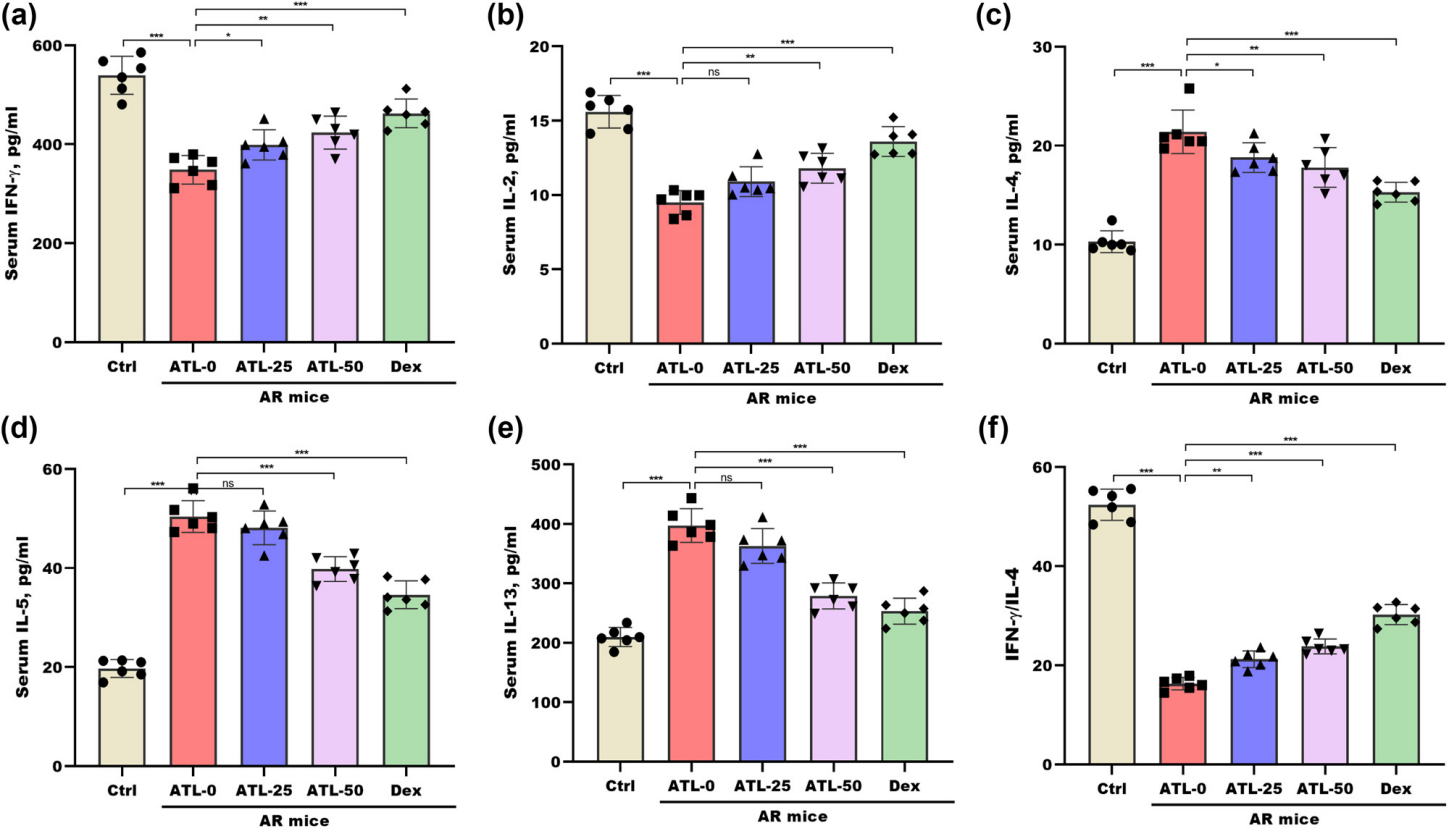
ATL-I improved the Th1/Th2 imbalance in AR mice. The serum concentrations of IFN-γ (a), IL-2 (b), IL-4 (c), IL-5 (d), and IL-13 (e) in the Ctrl, ATL-0, ATL-25, ATL-50, and Dex groups were measured via ELISAs. (f) The ratio of IFN-γ to IL-4 was calculated according to their concentrations. *p < 0.05, **p < 0.01, ***p < 0.001, ns, not significant.

The ability of ATL-I to improve the Th1/Th2 imbalance was next investigated by measuring the serum concentrations of Th1 and Th2 cytokines. Figure 3a and b shows that the serum IFN-γ and IL-2 levels were markedly lower in AR mice than in control mice, whereas these decreases were prevented by ATL-I. Conversely, the serum concentrations of IL-4, IL-5, and IL-13 were markedly increased in AR mice, whereas these changes were blocked by ATL-I (Figure 3c–e). Compared with that in normal mice, the IFN-γ-to-IL-4 ratio in AR mice was significantly lower, whereas this decrease was counteracted by ATL-I (Figure 3f).

**Figure 4:**
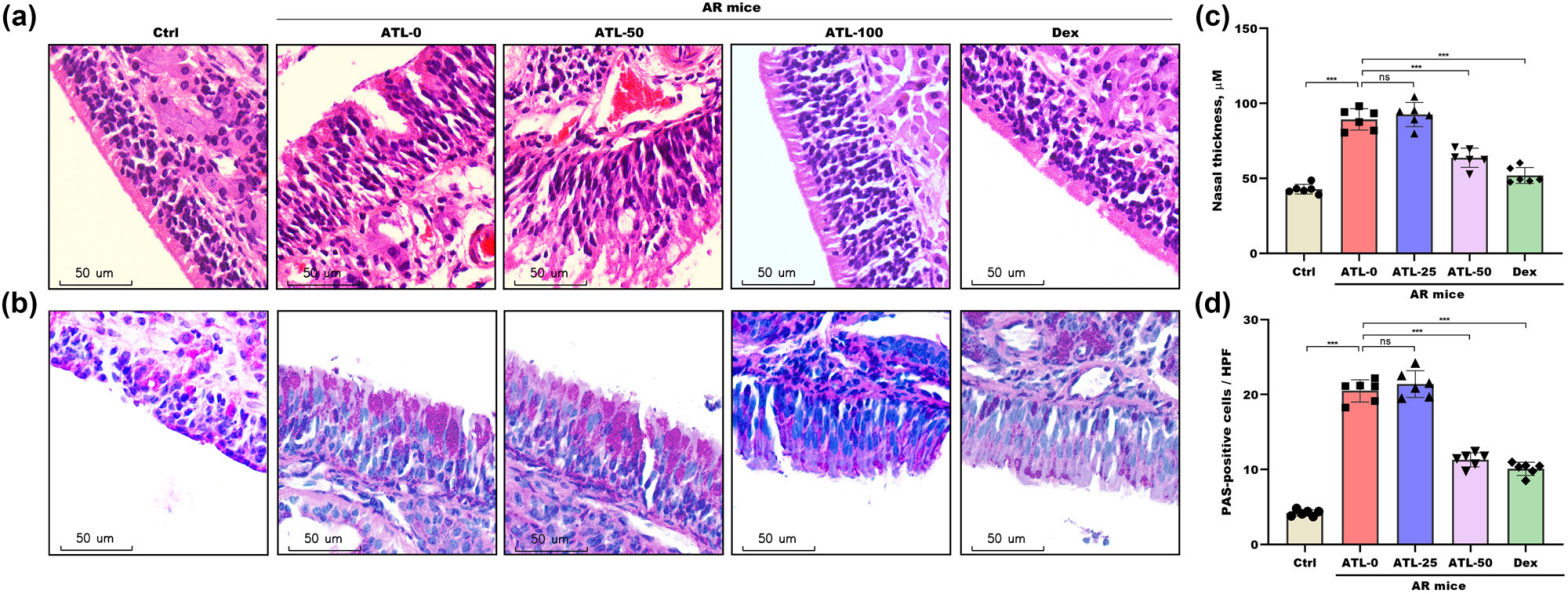
The roles of ATL-I in histological alterations. H&E (a) and PAS (b) staining of nasal tissues from the Ctrl group, the ATL-0 group, the ATL-25 group, the ATL-50 group, and the Dex group. Epithelial thickness (c) and goblet cell hyperplasia (d) were assessed in nasal tissues from the Ctrl group, the ATL-0 group, the ATL-25 group, the ATL-50 group, and the Dex group. **p < 0.01, ***p < 0.001, ns, not significant.

The impact of ATL-I on histological changes in the nasal mucosa was subsequently assessed. H&E staining revealed an orderly arrangement of epithelial cells and ciliated tissues in the normal nasal mucosa (Figure 4a). Conversely, the mucosal tissues from AR mice often displayed epithelial exfoliation and dilation of blood vessels in the lamina propria. These changes, however, were mitigated by ATL-I (Figure 4a). The epithelial thickness was significantly increased in AR mice, which was effectively prevented by ATL-I (Figure 4a and c). Compared with that of normal mice, the nasal mucosa of AR mice exhibited a marked increase in goblet cell hyperplasia, which was subsequently mitigated by ATL-I (Figure 4b and d).

### 3.3 ATL-I suppressed TLR4/NF-κB activation

**Figure 5:**
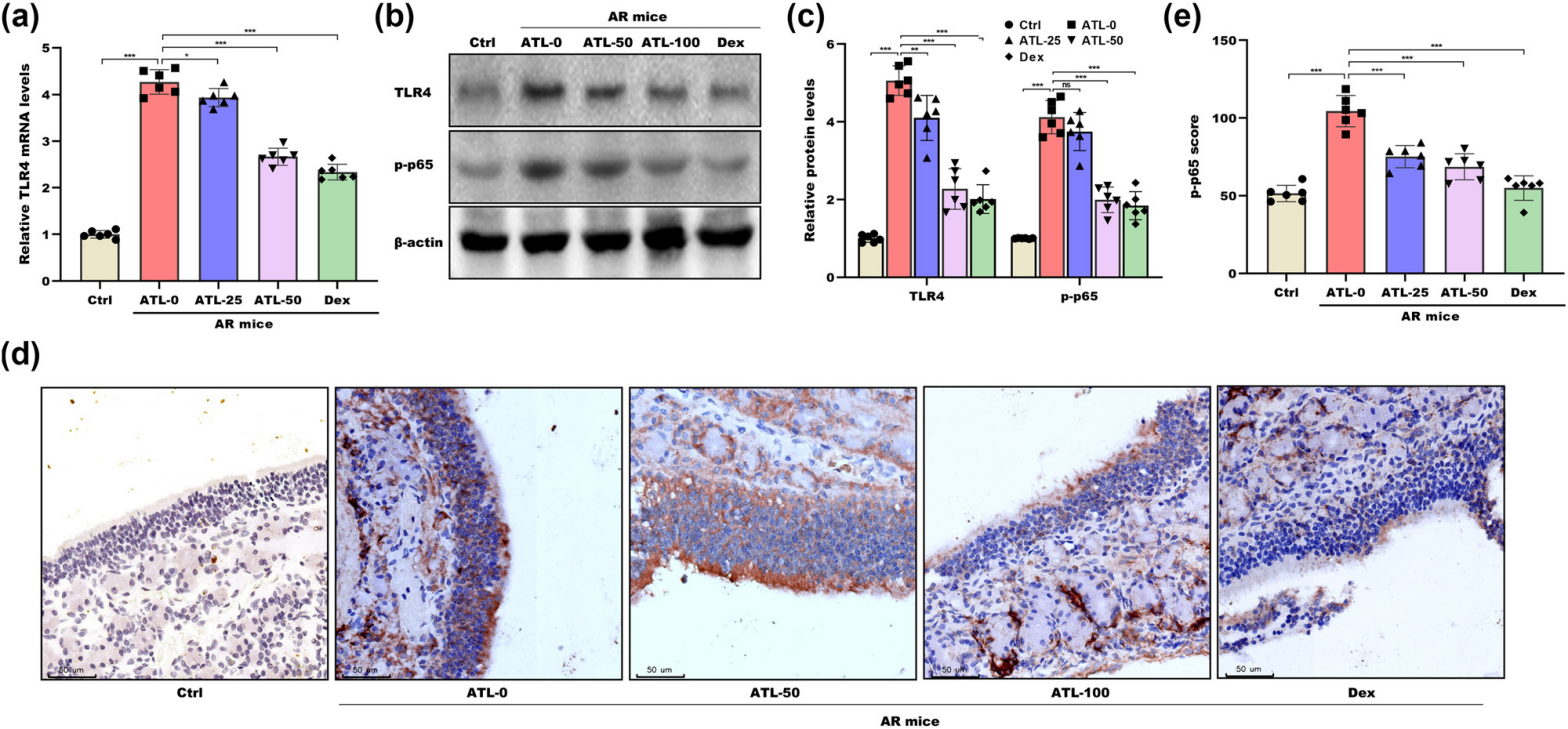
ATL-I suppressed the TLR4/NF-κB signalling pathway. (a) qRT-PCR analysis of TLR4 mRNA expression in nasal mucosal tissues from the Ctrl group, the ATL-0 group, the ATL-25 group, the ATL-50 group, and the Dex group. Western blot (b) and quantitative (c) analyses of TLR4 and p-p65 protein levels in nasal mucosal tissues from the Ctrl group, the ATL-0 group, the ATL-25 group, the ATL-50 group, and the Dex group. IHC (d) and quantitative (e) analysis of p-p65 protein levels in nasal mucosal tissues from the Ctrl group, the ATL-0 group, the ATL-25 group, the ATL-50 group, and the Dex group. *p < 0.05, **p < 0.01, ***p < 0.001, ns, not significant.

Accumulating evidence has verified that the TLR4/NF-κB pathway is commonly activated in the nasal mucosa of AR mice and that the inhibition of this signalling pathway is crucial for ameliorating allergic responses [7,30,31,32,33,34]. As shown in Figure 5a–c, significant increases in TLR4 mRNA and protein expression were observed in AR mice, which were effectively blocked by ATL-I. Furthermore, p-p65 levels were markedly increased in the nasal mucosa of AR mice, whereas these increases were prevented by ATL-I (Figure 5b and c). IHC analysis revealed that ATL-I prominently decreased p-p65 levels in the nasal mucosa of AR mice (Figure 5d and e). Consistent with previous reports [31,35], the TLR4 antagonist TAK-242 markedly inhibited NF-κB signalling activation (Figure S1a and b) and improved the Th1/Th2 imbalance in AR mice (Figure S1c–g). These data indicate that ATL-I inhibits the activation of TLR4/NF-κB signalling in AR mice, which ameliorates Th1/Th2 imbalance.

### 3.4 ATL-I suppressed NLRP3 inflammasome activation

**Figure 6:**
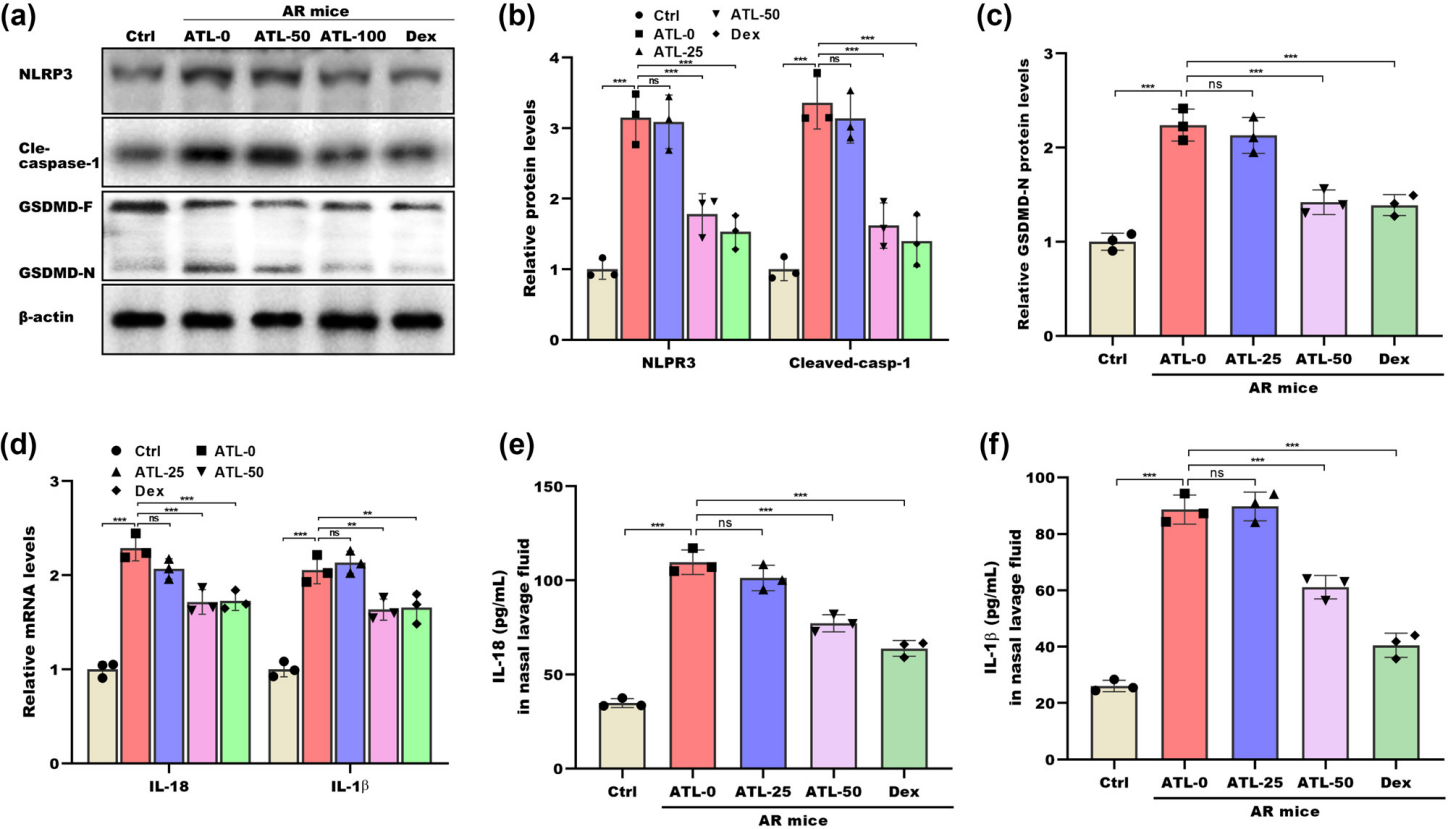
ATL-I suppressed the activation of the NLRP3 inflammasome. Western blot (a) and quantitative (b) and (c) analyses of the levels of the NLRP3, cleaved caspase-1, and GSDMD proteins in nasal mucosal tissues from the Ctrl group, the ATL-0 group, the ATL-25 group, the ATL-50 group, and the Dex group. (d) qRT-PCR analysis of IL-18 and IL-1β mRNA expression in nasal mucosal tissues from the Ctrl group, the ATL-0 group, the ATL-25 group, the ATL-50 group, and the Dex group. IL-18 (e) and IL-1β (f) levels in the nasal lavage fluid from the Ctrl, ATL-0, ATL-25, ATL-50, and Dex groups were measured by ELISAs. *p < 0.05, **p < 0.01, ***p < 0.001, ns, not significant.

The effects of ATL-I on alleviating NLRP3 inflammasome activation in the nasal mucosa were subsequently investigated. As shown in Figure 6a and b, NLRP3 and cleaved caspase-1 protein levels were markedly increased in AR mice compared with control mice, and ATL-I treatment reversed this process. More importantly, ATL-I decreased GSDMD cleavage in AR mice (Figure 6a and c). Compared with those of control mice, the nasal tissues of AR mice presented increased IL-18 and IL-1β levels. However, these increases were blocked by ATL-I (Figure 6d). ATL-I treatment also resulted in decreased release of IL-18 and IL-1β in the nasal lavage fluid of AR mice (Figure 6e and f).

## 4 Discussion

AR is a common allergic disease that affects approximately 30% of the world’s population [36]. An increasing number of reports have highlighted the effects of plant-derived compounds on the treatment of AR. For example, the extract of Osterici Radix has antiallergic properties [37], whereas *Nigella sativa* has been found to alleviate symptoms such as nasal mucosal congestion, nasal itching, runny nose, and sneezing [38]. Furthermore, methylwarifteine has been reported to reduce eosinophilia, the number of inflammatory cells, and NF-кB activation in granulocytes and lymphocytes [39]. ATL-I, a compound used to treat various diseases, has been studied for its potential role in AR, although its efficacy remains unconfirmed [14,15,16]. Several studies have indicated that ATL-I functions by regulating TLR4 and related signalling pathways. For example, ATL-I mitigates liver damage by inhibiting TLR4/mitogen-activated protein kinase (MAPK)/NF-κB signalling [40], increasing the chemosensitivity of cancer cells to paclitaxel through the regulation of TLR4/MyD88 signalling [41], and decreasing breast cancer progression via the inhibition of TLR4/NF-κB signalling [14]. Given the crucial role of TLR4/NF-κB signalling in allergic inflammation, this study aimed to explore whether ATL-I has a protective effect on AR. The results revealed that (i) ATL-I mitigated the clinical symptoms of AR mice; (ii) ATL-I ameliorated the Th1/Th2 imbalance in AR mice; (iii) ATL-I decreased TLR4/NF-κB signalling activation; and (iv) ATL-I suppressed NLRP3 inflammasome activation.

The Th1/Th2 imbalance is crucial in the development of AR. Plant-derived compounds exhibit therapeutic potential in treating AR by addressing the Th1/Th2 imbalance. For example, Ke et al. showed that quercetin partially mitigates AR symptoms by inhibiting the Th1/Th2 imbalance [42]. Similarly, bergapten modulates the Th1/Th2 ratio by regulating the levels of cytokines associated with these T-helper cells in an AR mouse model [43]. Nguyen et al. proposed *Artemisia gmelinii* extracts as potential therapeutic agents for AR because of their role in maintaining Th1/Th2 homeostasis [44]. While a few studies have revealed the role of ALT in improving the Th1/Th2 imbalance [45], the function of ATL-I in AR has yet to be defined. Here, we discovered that ATL-I plays a regulatory role in the Th1/Th2 imbalance by increasing the levels of Th1-related cytokines (IL-2 and IFN-γ) and decreasing the levels of Th2-related cytokines (IL-4, IL-5, and IL-13) in AR mice. Moreover, ATL-I improved the Th1/Th2 imbalance by inactivating the TLR4/NF-κB/NLRP3 pathway. This finding aligns with emerging studies that highlight the regulatory role of ATL-I in the TLR4/NF-κB pathway [14,40]. This study is the first to reveal the role of ATL-I in addressing Th1/Th2 imbalance by decreasing TLR4/NF-κB activation.

ATL-I alleviates AR symptoms by ameliorating the Th1/Th2 imbalance through the inhibition of TLR4/NF-κB/NLRP3 activation, suggesting that ATL-I is a potential therapeutic agent for AR. Nevertheless, this study has several limitations: (i) Given the significant roles of other signalling pathways, including Janus kinase (JAK)/signal transducer and activator of transcription (STAT), MAPK, and activator protein 1 (AP-1), in AR, investigating the regulatory effects of ATL-I on these pathways is essential. Xu et al. reported that chlorogenic acid treatment effectively mitigates allergic responses by regulating the TLR4/MAPK/NF-κB pathway [46]. Psoralen exerts anti-inflammatory effects in AR through inactivating the AP-1 signalling pathway [47]. ATL-I has been shown to decrease the levels of phosphorylated JAK2 and phosphorylated STAT3 [48]. Therefore, further investigation into the roles of ATL-I in these signalling pathways is warranted to fully elucidate its therapeutic potential in AR. (ii) It is necessary to further investigate the optimal administration route and dosage of ATL-I. Intranasal administration may be a suitable route, allowing targeted delivery to nasal tissues while minimizing systemic exposure. Compared to conventional antihistamines and corticosteroids, ATL-I may offer advantages such as fewer adverse effects or enhanced modulation of inflammatory pathways. However, further studies are needed to evaluate its long-term safety, optimal dosing strategies, and potential formulation challenges.

## 5 Conclusions

These results show that ATL-I alleviates allergic responses by inhibiting the TLR4/NF-κB/NLRP3 pathway, providing a promising therapeutic strategy for AR.

## Supplementary Material

Supplementary material
