# Supplementary material for: Atractylenolide I alleviates the experimental allergic response in mice by suppressing TLR4/NF-kB/NLRP3 signalling
Source: Open Life Sci. 2025 Aug 8;20(1):20251143. doi: 10.1515/biol-2025-1143 (PMC12355362; doi:10.1515/biol-2025-1143)
Supplement: Supplementary material [file biol-2025-1143-sm.pdf]

Supplementary material

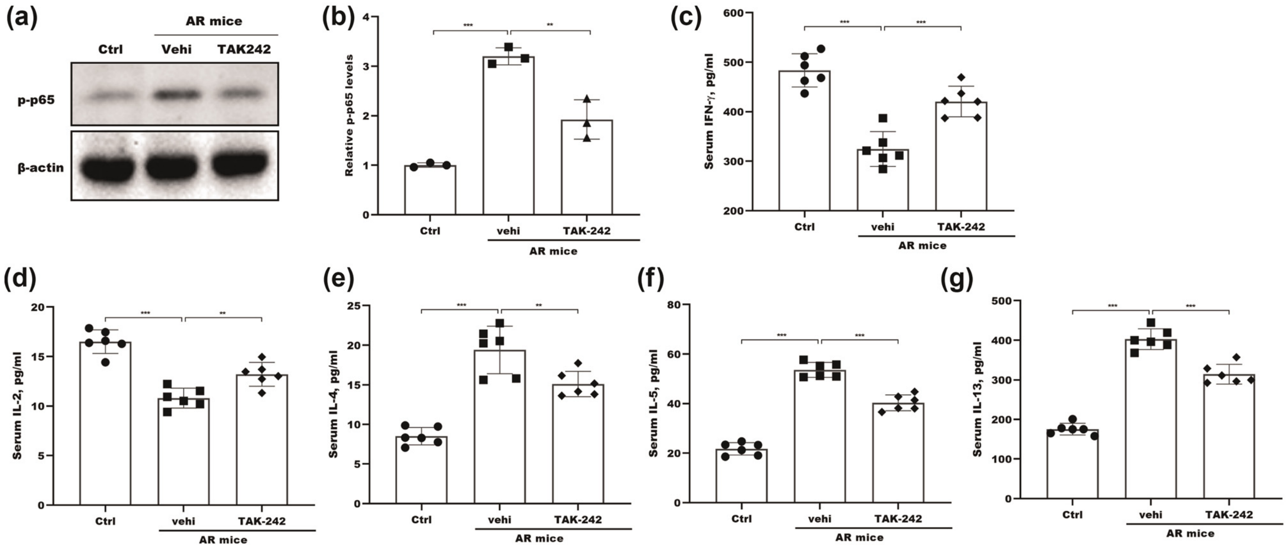

Figure S1: Western blot (A) and quantitative (B) analyses of p-p65 protein levels in nasal mucosal tissues from the control mice, AR mice, and TAK242-treated AR mice. The serum concentrations of IFN-γ (C), IL-2 (D), IL-4 (E), IL-5 (F), and IL-13 (G) in the control mice, AR mice, and TAK242-treated AR mice were measured via ELISAs. ** $p < 0.01$, *** $p < 0.001$.

Table S1: QRT-PCR primers used in the study

Gene symbol	Sense, 5′–3′	Antisense, 5′–3′
TLR4	CTGGCATCATCTTCATTGTCCT	TGCCGTTTCTTGTTCTTCCTCT
IL-18	CCTTTGAGGCATCCAGGACAA	ACAGCCAGTGTTCAGTCAGC
IL-1β	CCTGTGTCTTTCCCGTGGAC	GCACGAGGCTTTTTTGTTGTTC
β-actin	GGAGTACGATGAGTCCGGC	GTGTAAAACGCAGCTCAGTAACA
